# Immunization with a multisubunit vaccine induces immunological memory and cross-protective immunity against heterologous *Chlamydia* serovars

**DOI:** 10.3389/fcimb.2026.1941899

**Published:** 2026-09-17

**Authors:** Fnu Medhavi, Shakyra Richardson, Roshan Pais, Awa Sore, Yusuf Omosun, Francis O. Eko

**Affiliations:** 1 Department of Microbiology, Biochemistry and Immunology, Morehouse School of Medicine, Atlanta, GA, United States; 2 School of Basic and Applied Sciences, Dayananda Sagar University, Bengaluru, Karnataka, India

**Keywords:** chlamydia trachomatis, cross-protection, immunological memory, mucosal immunity, rVCG-MECA, subunit vaccine, systemic immunity

## Abstract

We previously reported that intramuscular (IM) and intranasal (IN) immunization with a recombinant *Vibrio cholerae* (rVCG)-based multiepitope subunit vaccine (rVCG-MECA) elicited robust long-lasting, cellular, and humoral immune effectors that protected mice against transcervical (TC) challenge infection with the homologous *C. trachomatis* (CT) serovar D. In this study, we evaluated the ability of this vaccine to confer cross-protection against primary and secondary infection with the heterologous CT serovars F and J and induce cross-reactive immunological memory following reinfection. Thus, groups of female C57BL/6 mice were immunized and boosted twice IM or IN 2 weeks apart with rVCG-MECA or PBS or unimmunized (naïve) controls. Vaccine immunization elicited serum immunoglobulin G (IgG) antibodies with moderate to high avidity. Four weeks postimmunization, mice were challenged transcervically with 1 × 10^6^ IFU of CT serovar F or J and rechallenged 10 weeks after initial challenge. The humoral and cell-mediated immune responses elicited at weeks 1 and 4 following rechallenge were evaluated. Irrespective of route of immunization, all immunized mice had significantly (*p* < 0.0001) reduced genital bacterial burden beginning as early as day 3 following primary challenge infection with the heterologous serovars F and J and completely the infection by day 21. However, PBS control mice still shed high numbers of *Chlamydia* at this time point. Also, mice rechallenged with serovars F and J 70 days after the primary challenge completely cleared the secondary infection by day 15. Furthermore, rechallenge of mice with serovars F and J resulted in the elicitation of high levels of chlamydial-specific IgG2a and IgA antibodies in sera and vaginal secretions as well as the production of high concentrations of the T helper-1 (Th1) cytokines, interferon-γ (IFN-γ) and interleukin-12 (IL-12), and low levels of the Th2 cytokine, IL-4, in the spleen and draining lymph nodes. These findings demonstrate that rVCG-MECA induces durable, cross-protective immunity against multiple CT serovars, indicating its potential as a promising universal vaccine candidate for the prevention of genital chlamydial infections.

## Introduction

*Chlamydia trachomatis* (CT) is the leading cause of bacterial sexually transmitted infections (STIs) globally, with an estimated 129 million new cases annually (CDC, 2022; Murray et al., 2022). While many infections are asymptomatic, untreated cases can lead to severe reproductive complications in women, including pelvic inflammatory disease (PID), ectopic pregnancy, chronic pelvic pain, and infertility (Bebear and de Barbeyrac, 2009; Paavonen, 2012; Rowley et al., 2019). Despite its global burden and decades of research, there is currently no licensed vaccine against CT, underscoring the urgent need for effective immunization strategies (Igietseme et al., 2011; de la Maza et al., 2017; Phillips et al., 2019). The major obstacle in vaccine development lies in the high antigenic variability among CT serovars, each exhibiting different tissue tropisms, immune evasion mechanisms, and disease outcomes (de la Maza et al., 2017; Brunham, 2018). Of the 19 immunologically identified CT serovars (Ser), 8 (D, E, F, G, H, I, J, and K) are implicated in urogenital infections worldwide, while serovars L1–L3 cause lymphogranuloma venereum and Ser A, B, Ba, and C are implicated in trachoma (Brunelle and Sensabaugh, 2006; Darville and Hiltke, 2010). Ser D, E, and F are the three most frequently isolated urogenital serovars globally (Bandea et al., 2001) while Ser A and B are the most common ocular isolates. Based on mouse serum cross-reactivity and cross-protection studies and ompA phylogeny (Mordhorst, 1963; Alexander et al., 1967; Stothard et al., 2003; Brunelle and Sensabaugh, 2006), the eight urogenital (D–K) and three trachoma (A, B, and C) Ser are grouped into three distinct antigenicity clusters or complexes: cluster 1 (or B complex) (Ser B, D, and E), cluster 2 (or Intermediate complex) (Ser F and G), and cluster 3 (or C complex) (Ser A, C, H, I, J, and K), with serovars E, F, and J being the senior members of each cluster, respectively. Murine model studies established that infection-induced protection is restricted to the serovars within the same cluster; Ser D- and E-induced immunity cross-protected each other as did serovars F and G while Ser J-induced immunity protected against the other members in the cluster (Tuffrey et al., 1992; Ramsey et al., 1999; Ramsey et al., 2000).

Epidemiological studies indicate that the protective immunity afforded following natural chlamydial infection declines with age, suggesting that partial immunity develops over time, though reinfection remains common (Batteiger et al., 2010). Vaccines targeting a single antigen such as the major outer membrane protein (MOMP) have been shown to afford homologous serovar protection but fail to protect against heterologous serovars (Igietseme and Murdin, 2000). Thus, recent vaccine strategies have turned to a multivalent subunit approach. In this regard, we previously demonstrated the ability of a multisubunit vaccine to induce cross-protective chlamydial immunity and establish the possibility of developing a broadly efficacious CT vaccine (Eko F. O. et al., 2011). Recently, a MOMP-based vaccine construct targeting the variable domain 1 (VD1) region that was designed to cover multiple MOMP serovars demonstrated broad neutralizing antibody activity and protection across multiple CT C-complex serovars in mouse models (Olsen et al., 2021). Moreover, longitudinal preclinical work with the CTH522/CAF^®^01 vaccine illustrated the importance of durable cross-reactive immune responses (Olsen et al., 2024).

Recent advances in subunit vaccine development have focused on utility of delivery systems that are safe, non-replicating, and support multiple antigen delivery, including the *Vibrio cholerae* ghost (VCG) delivery platform. VCG are non-living, non-denatured *V. cholerae* cell envelopes that are devoid of cytoplasmic contents while maintaining their cellular morphology and surface antigenic structure, including pathogen-associated recognition receptors (Eko et al., 2000; Eko et al., 2003). The VCG platform represents a flexible and capacious delivery vehicle that can simultaneously target multiple antigens to the immune system. We recently reported that intramuscular (IM) and intranasal (IN) immunization with a VCG-based multiepitope *Chlamydia* subunit vaccine, rVCG-MECA, elicited robust long-lasting, cellular, and humoral immune effectors that protected mice against transcervical (TC) challenge infection with the homologous CT serovar D (Medhavi et al., 2025). Since different chlamydial serovars predominate in different geographic regions, a successful vaccine should prevent infection by diverse CT serovars (Eko et al., 2004; Vasilevsky et al., 2014; Lanfermann et al., 2021).

In this study, we evaluated the ability of rVCG-MECA to confer cross-protection against primary and secondary infection with the heterologous CT serovars F and J and induce cross-reactive immunological memory responses following reinfection of immunized mice with heterologous CT serovars. The results revealed that immunization with the rVCG-MECA vaccine confers cross-protection against the heterologous CT serovars F and J and induces robust cross-reactive mucosal and systemic memory immune responses following reinfection with heterologous CT serovars. These results indicate the potential of rVCG-MECA as a promising universal vaccine candidate for the prevention of genital CT infections.

## Materials and methods

### Ethics statement

All animal experiments described in this study were conducted in accordance with the National Institutes of Health Guide for the Care and Use of Laboratory Animals. The experimental protocols were reviewed and approved by the Institutional Animal Care and Use Committee of Morehouse School of Medicine (Animal Welfare Assurance No. A3381-01; IACUC Approval No. 19-09).

Female C57BL/6J mice, 6 weeks of age (The Jackson Laboratory, stock no. 000664), were maintained in the Morehouse School of Medicine Center for Laboratory Animal Resources under specific pathogen-free conditions. Animals were provided with unrestricted access to food and water, housed under a 12-h light/dark cycle, and allowed to acclimate for a minimum of 7 days prior to the initiation of experimental procedures.

To minimize pain and distress, all immunizations, sample collections, and challenge infections were performed under isoflurane inhalation anesthesia administered in an induction chamber using 2%–4% isoflurane in oxygen within a fume hood, and anesthetic depth was monitored throughout each procedure by assessing lack of withdrawal to a toe pinch. At experimental endpoints, mice were euthanized using carbon dioxide (CO_2_) asphyxiation, followed by cervical dislocation in alignment with the American Veterinary Medical Association (AVMA) Guidelines for the Euthanasia of Animals.

### *Chlamydia* stocks and antigens

In-stock preparations of CT serovars D (UW-3/Cx), F (IC-Cal-3), and J (UW-36/Cx) used in this study were originally obtained from the Centers for Disease Control and Prevention (CDC), Atlanta, GA, and were previously titrated on HeLa-229 cell (ATCC Cat# CCL-2.1, RRID: CVCL_1276) monolayers followed by purification of chlamydial elementary bodies (EBs) over renografin density gradient centrifugation. The purified EBs were then resuspended in sucrose-phosphate-glutamate (SPG; 220 mM sucrose, 12.5 mM NaH_2_PO_4_, and 4 mM L-glutamic acid; pH 7.5) buffer and stored at −80°C until used. The number of chlamydial inclusion-forming units (IFU) was quantified by titration in HeLa-229 cells. Chlamydial antigens were prepared by exposing purified EBs to UV radiation for 3 h on ice and stored at −80°C until used.

### Design and construction of the multiepitope CT antigen

The design and construction of the multiepitope CT vaccine has previously been described (Tanner et al., 2024; Medhavi et al., 2025). Briefly, linear T- and B-cell epitopes were predicted from five CT serovar D outer membrane proteins (PmpD, PmpC, PmpG, PorB, and OmcB) that are conserved across the eight urogenital CT serovars and were previously shown to be immunogenic (Brunham and Peeling, 1994; Kubo and Stephens, 2000; Yu et al., 2014; Vasilevsky et al., 2016). Twenty linear epitopes (366 amino acids) selected on the basis of high scoring physicochemical properties were fused together using P_2_G_3_ linkers together with the A1 subunit (194 amino acids) of cholera toxin (CTA1) adjuvant at the N-terminal region via an EAAAK linker (MECA) (**Figure 1A**). The 660-amino-acid MECA sequence was reverse-translated and inserted into the periplasmic targeting vector, pFLAG-CTS to generate pCT-MECA.

**Figure 1:**
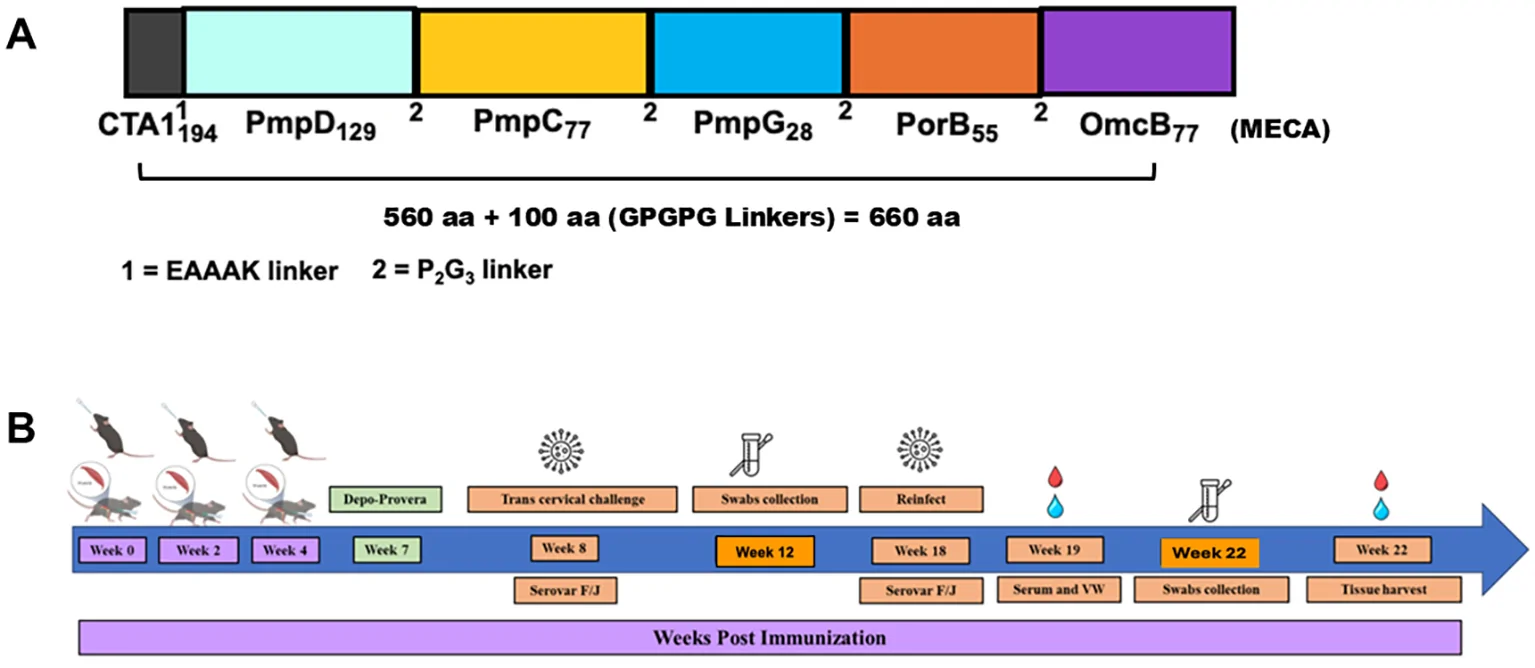
Design of the multiepitope CT antigen (MECA) **(A)** and schematic diagram of the experimental protocol **(B)** outlining the immunization, sample collection, challenge, and rechallenge schedules.

### Production of the rVCG-MECA vaccine

Competent *V. cholerae* O139 strain V912 (a laboratory strain originally obtained from Dr. Paul Manning, University of Adelaide, Australia) transformed with plasmid pCT-MECA was co-transformed with the lysis plasmid pDKLO1 and grown in brain heart infusion (BHI) broth at 37°C to mid-log phase. Production of the rVCG-MECA vaccine was carried out by protein E-mediated lysis essentially as described previously (Eko et al., 2003; Medhavi et al., 2025). At the end of lysis, cultures were harvested, washed with phosphate buffered saline (PBS), and lyophilized. Lyophilized VCGs were weighed, and the number of CFU per milligram of VCG was estimated based on the total number of CFU in the culture medium at the highest absorbance attained before lysis. VCG preparations were stored at room temperature or refrigerated at 8°C until used. The vaccine candidates consisted of recombinant VCG expressing the multiepitope chlamydial antigen (MECA) or live chlamydial EBs or PBS control.

### Immunization and challenge

Female C57BL/6 mice (*n* = 12 per group) were immunized and boosted twice, 2 weeks apart via the IM or IN route with 1.5 mg of lyophilized rVCG-MECA in 30 ul of PBS or a single IN dose of live CT containing 10^6^ IFU of Ser D (positive control) on week 4 as shown in **Figure 1B**. Naive unimmunized or PBS-alone groups served as negative controls.

Four weeks (week 8) after the last immunization, the mice were challenged transcervically (TC) with 10^6^ IFU of live CT serovar F or J elementary bodies (Live CT EBs) using a non-surgical embryo transfer (NSET) device for mice (ParaTechs Corp., Lexington, KY) as previously described (Pal et al., 2018; Rajeeve and Sivadasan, 2020). All infections were delivered in 10 μL of SPG buffer. One week prior to challenge (week 7), each mouse was injected with 2.5 mg of medroxyprogesterone (Depo Provera; Pharmacia UpJohn Co., Kalamazoo, MI) subcutaneously to synchronize the estrous cycle and increase mouse susceptibility to infection. Briefly, the NSET adaptor was inserted into the vaginal vault and the NSET needle was then inserted through the adaptor till it reached the cervix of the mice. Using a P20 micropipette, the CT challenge dose in 10 μL of SPG buffer was delivered to the uterine fundus. The mice were monitored for several minutes for any adverse signs and then returned to the cage. Cervicovaginal swabs were collected on week 12 every 6 days for 30 days to assess genital CT burden as reported previously (Pal et al., 2018; Rajeeve and Sivadasan, 2020; Medhavi et al., 2025; Pal et al., 2025). Ten weeks after the primary infection (week 18), a time when previously infected mice usually become susceptible to reinfection (Tuffrey et al., 1984; Tuffrey et al., 1990; Morrison and Caldwell, 2002; Rank and Whittum-Hudson, 2010), the mice were rechallenged TC with 10^6^ IFU of live CT serovar F or J and cervicovaginal swabs were collected on week 22 every 6 days for 30 days.

### Enumeration of chlamydial inclusion-forming units

To monitor chlamydial burden, chlamydial IFU were quantified from cervicovaginal swab samples collected every 6 days for 30 days following the challenge infection. The swabs were thawed on ice and vortexed, and 300 μL of each sample was used to infect confluent McCoy cell monolayers cultured in 24-well plates. Plates were centrifuged at 1,400 rpm at 4 °C for 1 h to facilitate infection and then incubated at 37 °C for 1 h. The inoculum was removed and replaced with Iscove’s Modified Dulbecco’s Medium (IMDM) containing 50 μL of cycloheximide per 50 mL of IMDM supplemented with 10% FBS and incubated for 48 h. Following incubation, cells were fixed with ethanol and stained using the Thermo Scientific PathoDx Chlamydia culture confirmation kit, according to the manufacturer’s recommendations (Fisher Scientific, Catalog No. R62210). Briefly, cells were washed with PBS, incubated with staining reagent (fluorescein-conjugated murine monoclonal antibodies targeting the chlamydial MOMP, Evans Blue counterstain), and visualized by indirect immunofluorescence microscopy. The level of infection was assessed by enumerating the number of chlamydial IFU from each swab by indirect immunofluorescence and the mean number of IFU at each time point was calculated.

### Assessment of antigen-specific antibody avidity

The avidity of CT-specific serum immunoglobulin G (IgG) and IgG2c antibodies collected at weeks 2 and 4 postimmunization was evaluated using a modified enzyme-linked immunosorbent assay (ELISA) protocol (Almanzar et al., 2013) incorporating antibody standards and ammonium thiocyanate (NH_4_SCN) as described previously (Medhavi et al., 2025). Briefly, a 96-well plate was coated with 10 μg/mL of CT antigen (Ser F or J) in sodium carbonate buffer and incubated overnight at 4°C. After washing, the wells were blocked and incubated with serum samples at 37°C for 1 h followed by incubation with 2 M NH_4_SCN for 20 min. Following additional washes, horseradish peroxidase (HRP)-conjugated goat anti-mouse secondary antibody (diluted 1:5,000) was added, followed by ABTS substrate, and incubated for 1 h at 37°C. The absorbance was measured at 450 nm. The data were expressed as percent relative avidity index calculated by dividing the mean antibody concentration with NH_4_SCN by the concentration without NH_4_SCN multiplied by 100. Avidity was classified as high (≥50%), intermediate (30%–49%), or low (below 29%) based on the index values.

### Assessment of humoral memory responses

Serum and vaginal wash samples were collected at weeks 1 and 4 following reinfection (week 19) and pooled for each group to assess the *Chlamydia*-specific mucosal and systemic memory recall IgG2c and IgA antibody responses using a standard ELISA described previously (Macmillan et al., 2007). In brief, Maxisorb 96-well plates (Costar) were coated overnight with Ser F or J chlamydial antigen (10 μg/mL) in 100 μL of PBS at 4 °C and then blocked with 1% bovine serum albumin (BSA) containing 5% goat serum in PBS. Serum (100 μL) or vaginal wash (50 μL) samples in twofold serial dilutions were added per well and the plates were incubated with 100 μL of HRP-conjugated goat anti-mouse IgA or IgG2c isotypes (Southern Biotechnology Associates, Inc., Birmingham, AL) for 1 h. The plates were then developed by the addition of 2,2-azino-bis (3-ethylbenzthiazoline-6- sulfonic acid) (ABTS) (Sigma-Aldrich, St. Louis, MO, USA). The optical density was measured at 490 nm on a Microplate reader (Molecular Devices, Sunnyvale, CA, USA). Antibody concentrations were calculated from a standard curve generated simultaneously and display data sets as mean concentrations (ng/mL) ± SD from triplicate assays.

### Detection of cytokine production

Four weeks after reinfection (week 22), the mice were euthanized and spleens (SPL) and the iliac lymph nodes draining the genital tract (DLN) were harvested and pooled for each group. Single-cell suspensions were obtained from SPL and DLN using the gentleMACS Dissociator in combination with the Midi magnetic bead-activated cell sorting (MidiMACS) purification system and CD4 T cell-specific MACS microbeads (Miltenyi Biotech, Auburn, CA). The purity of the CD4^+^ T-cell population was determined to be at least >95% by flow cytometry. A pool of splenocytes isolated from naive mice and treated with mitomycin C (25 μg/10^7^ cells) for 20 min was used as a source of antigen-presenting cells (APCs). The purified CD4^+^ T cells were plated in quadruplicates at 2 × 10^5^ cells/well in 96-well tissue culture plates coated with 10 μg/mL UV-irradiated CT EBs Ser F or J (chlamydial antigen) and cultured with APCs (2 × 10^5^) for 5 days. Control cultures contained APCs and T cells without antigen. Culture supernatants were then harvested, and the amount of *Chlamydia*-specific cytokines [interferon-γ (IFN-γ), interleukin-12 (IL-12), IL-4, and IL-10] secreted by CD4 T cells was assessed using the Bio-Plex cytokine assay kit in combination with the Bio-Plex Manager software (Bio-Rad, Hercules, CA). The concentration of the cytokines in each sample was obtained by extrapolation from a standard calibration curve generated simultaneously. Data were calculated as the mean values (± SD) for triplicate cultures for each experiment. Parallel unstimulated cultures served as negative controls.

### Statistical analysis

Statistical analyses were performed with the GraphPad Prism 10 package (GraphPad Software, Inc., La Jolla, CA, USA) on a Mac computer. Statistical differences between groups were evaluated by one-way analysis of variance (ANOVA) with Tukey’s post-multiple comparison test. Statistical significance was determined at *p*-values ≤ 0.05, 0.01, 0.001, or 0.0001.

## Results

### Avidity of antigen-specific serum IgG and IgG2c antibodies

The avidity of CT-specific IgG and IgG2c antibodies elicited in serum following immunization was evaluated by a modified antibody ELISA. The results showed moderate avidity IgG antibodies against CT Ser F were elicited in serum of IM immunized mice at weeks 2 and 4 postimmunization (**Figure 2A**). However, while the avidity of IgG antibodies elicited in serum of IN immunized mice remained moderate at week 2, high-avidity antibodies (50%) were elicited 4 weeks postimmunization (**Figure 2A**). While the avidity of the T helper-1 (Th1)-associated IgG2c antibodies elicited at 2 and 4 weeks after IM immunization was moderate, the avidity of this antibody isotype elicited after IN immunization was high at both time points (**Figure 2B**). Also, the avidity of IgG2c antibodies elicited after IN mucosal immunization was significantly higher (65%) compared to those elicited after IM immunization (46%) at weeks 2 (*p* < 0.01) and 4 (*p* < 0.05) postimmunization (**Figure 2B**). In contrast, the relative avidity of IgG and IgG2c antibodies induced in mice following immunization with PBS (negative control) remained at baseline levels (20%) at both time points (**Figure 2**).

**Figure 2:**
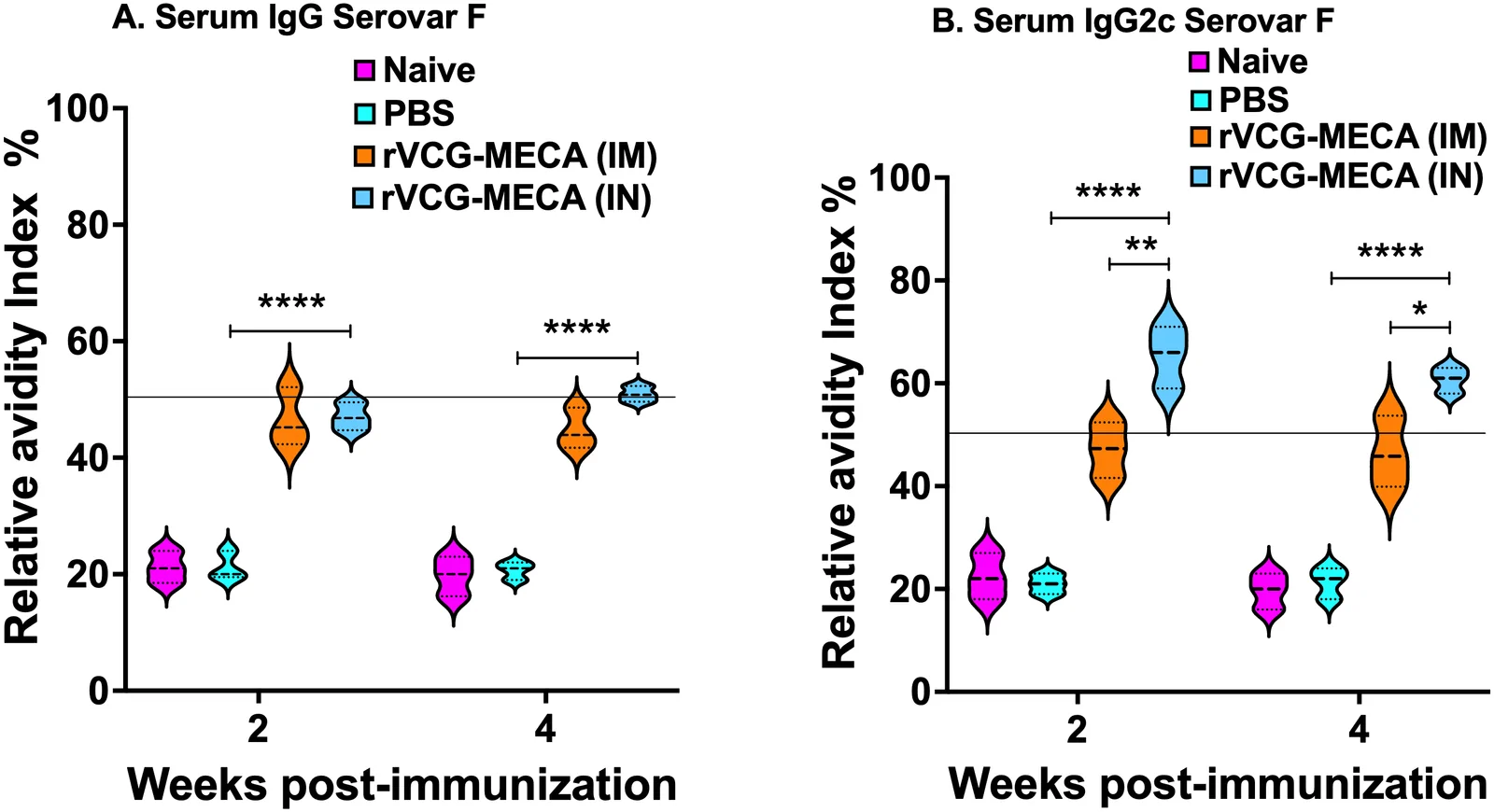
Avidity of serum IgG and IgG2c antibodies against CT Ser F after IM and IN immunization. The avidity of CT-specific IgG and IgG2c antibodies in serum samples from each immunization group was evaluated at 2 and 4 weeks postimmunization using a modified ELISA incorporating ammonium thiocyanate (NH_4_SCN). The results were generated simultaneously with a standard curve, and data sets, corresponding to absorbance values, were calculated as mean concentrations (ng/mL) ± SD of triplicate cultures for each experiment. The relative avidity index was calculated and displayed as a percentage of the ratio of the antibody concentration of samples treated with NH_4_SCN and that of untreated samples. The data, from one of two experiments with similar results, show the percent relative avidity index of serum IgG **(A)** and IgG2c **(B)** antibodies. Significant differences between experimental groups were compared by one-way ANOVA with Tukey’s post-multiple comparison test at *p < 0.05, **p < 0.01, and ****p < 0.0001.

Furthermore, while the avidity of serum IgG antibodies elicited in IM immunized mice against CT Ser J remained moderate at 2 and 4 weeks postimmunization, high-avidity IgG antibodies were elicited in the serum of IN immunized mice at both time points (**Figure 3A**). The avidity of these antibodies was significantly higher (*p* < 0.01) compared to those elicited after IM immunization (**Figure 3A**). Also, while the avidity of serum IgG2c antibodies elicited in IM immunized mice remained moderate 2 weeks postimmunization and were significantly lower (*p* < 0.001) compared to those elicited after IN immunization, the avidity of these antibodies had increased to high and were comparable to those elicited after IN immunization by week 4 (**Figure 3B**). As with Ser F, the avidity of serum IgG and IgG2c antibodies elicited in mice following immunization with PBS (negative control) remained at baseline levels (20%) at both time points (**Figure 3**). These findings indicate that the Th1-associated IgG2c antibody isotype elicited following IN immunization with rVCG-MECA have higher binding capacity compared to those induced by IM immunization.

**Figure 3:**
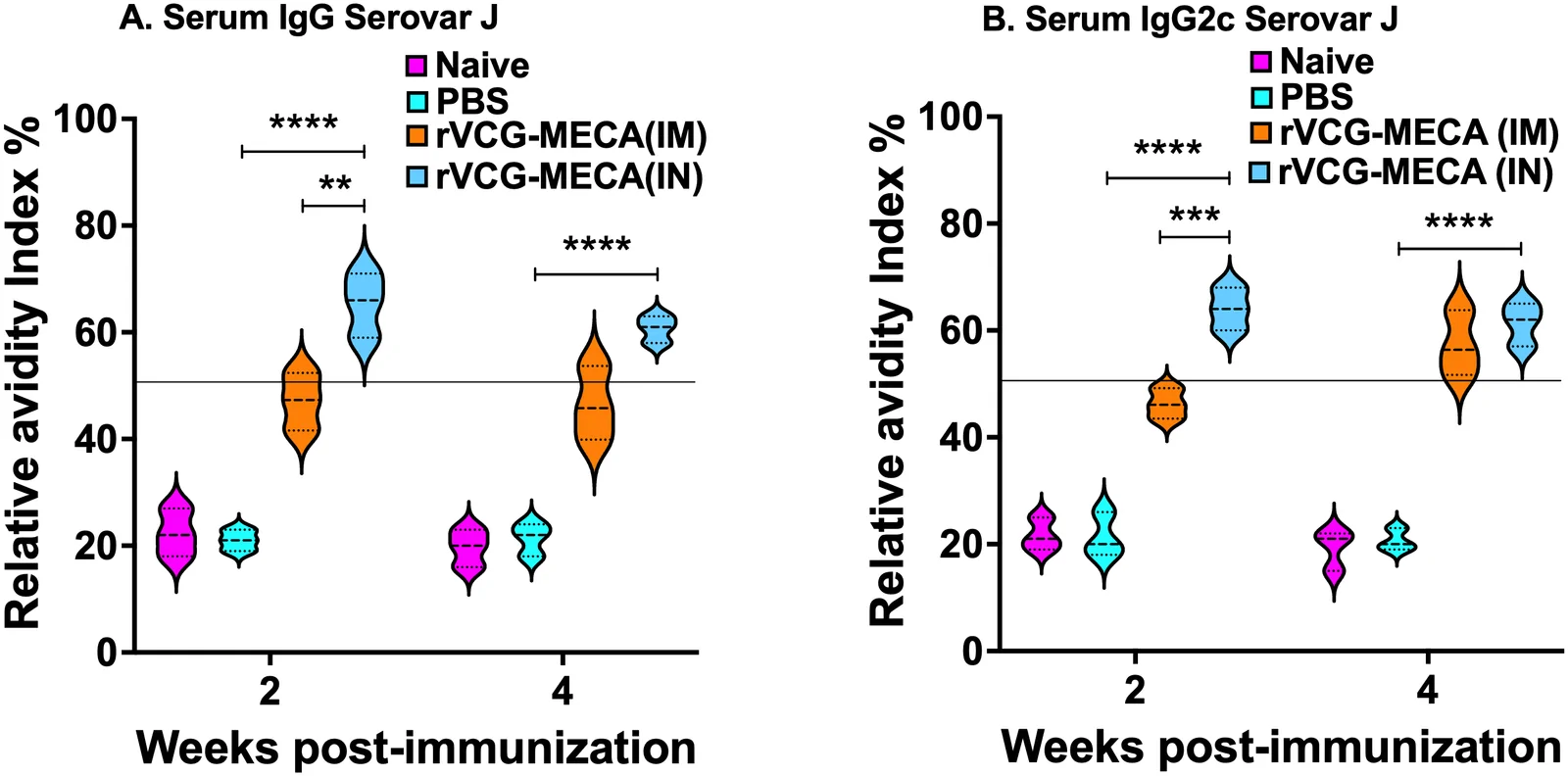
Avidity of serum IgG and IgG2c antibodies against CT Ser J after IM and IN immunization. The avidity of CT-specific IgG and IgG2c antibodies in serum samples from each immunization group was evaluated at 2 and 4 weeks postimmunization using a modified ELISA incorporating ammonium thiocyanate (NH_4_SCN). The results were generated simultaneously with a standard curve, and data sets, corresponding to absorbance values, were calculated as mean concentrations (ng/mL) ± SD of triplicate cultures for each experiment. The relative avidity index was calculated and displayed as a percentage of the ratio of the antibody concentration of samples treated with NH_4_SCN and that of untreated samples. The data, from one of two experiments with similar results, show the percent relative avidity index of serum IgG **(A)** and IgG2c **(B)** antibodies. Significant differences between experimental groups were compared by one-way ANOVA with Tukey’s post-multiple comparison test at **p < 0.01, ***p < 0.001, and ****p < 0.0001.

### Induction of cross-protection against primary challenge with live CT serovars F and J

To evaluate vaccine-induced cross-protection, immunized and control mice were challenged transcervically with CT serovar F or J, 4 weeks after the last immunization (**Figure 1B**). *Chlamydia* burden was monitored by periodic cervicovaginal swabbing of infected mice and inclusions in HeLa cell monolayers were enumerated by indirect immunofluorescence. The level of protection conferred by the rVCG-MECA vaccine was compared to that conferred by IN immunization with live CT Ser F or J (positive control) and IM immunization with PBS (negative control). The result showed that as early as 3 days following challenge, rVCG-MECA vaccine-immunized mice had significantly (*p* < 0.0001) reduced chlamydial burden compared to PBS-immunized mice and comparable to those of live CT-immunized mice (positive control), irrespective of route of vaccine administration or challenge serovar (**Figure 4**). Both routes of immunization also resulted in significantly (*p* < 0.0001) reduced chlamydial shedding from day 9 through day 15 compared to PBS controls. By day 21 after the challenge infection, all the rVCG-MECA vaccine-immunized and live CT EB-immunized mice had completely resolved the infection while the PBS-immunized mice continued to shed bacteria up to day 30 postchallenge. The result indicates that immunization with the rVCG-MECA vaccine conferred cross-protective immunity against serovars F and J comparable to that conferred by immunization with live CT.

**Figure 4:**
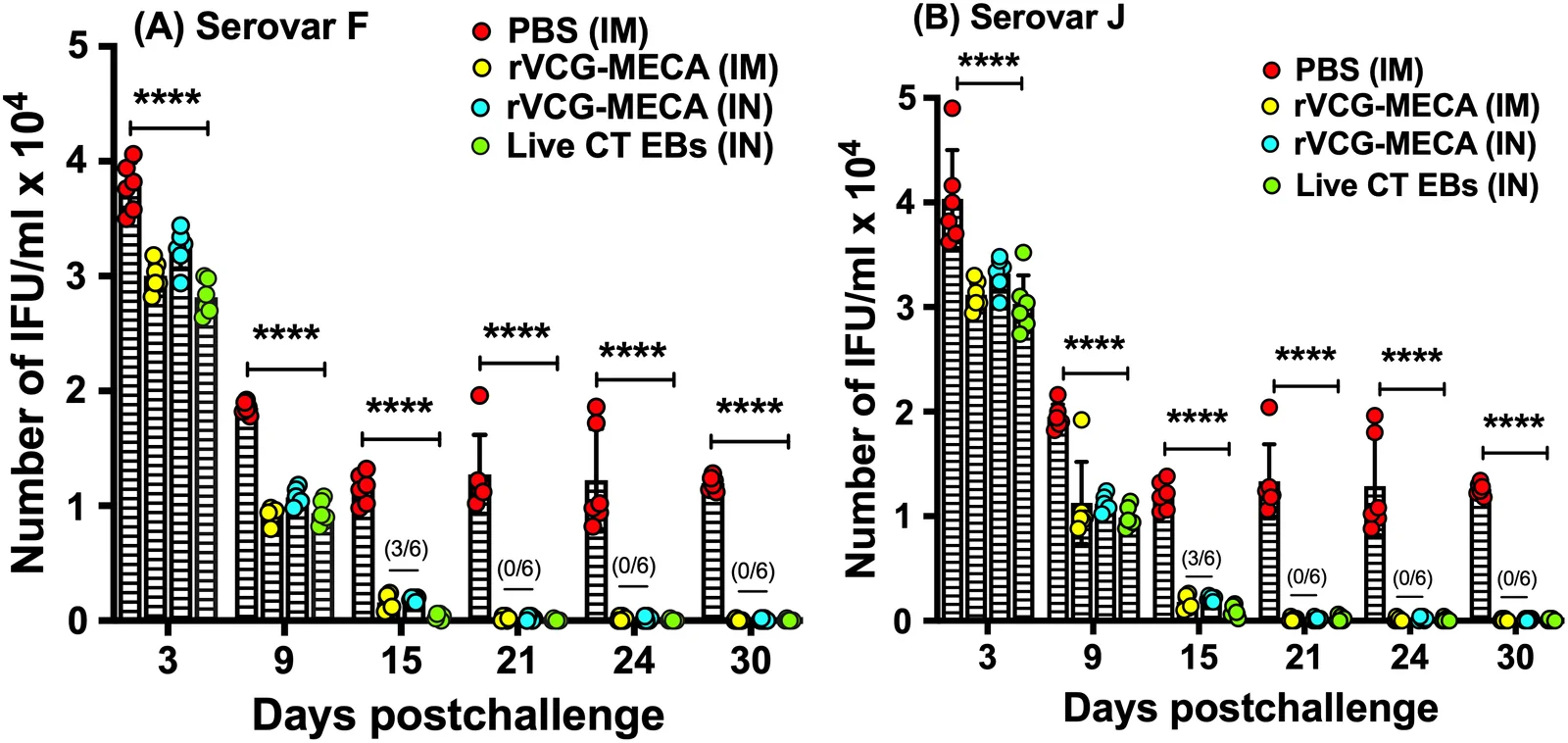
Cross-protection of rVCG-MECA vaccine-immunized mice against heterologous challenge with CT serovars F and J. Groups of mice were immunized and boosted twice IM or IN as described in the Materials and Methods section. Cross-protection against heterologous challenge with 10^6^ IFU of live CT Ser F **(A)** or J **(B)** was evaluated 4 weeks after the last immunization and CT burden was monitored by enumeration of chlamydial IFU from cervicovaginal swabs. Each data point represents the individual number of recoverable IFU/mL from each mouse (distinct colored circle) and the mean IFU for each group (represented by each column). Numbers in parentheses represent the number of mice with positive cultures at each time point. The differences between vaccine groups were compared by two-way ANOVA with Tukey’s post-multiple comparison test at ****p < 0.0001.

### Induction of cross-reactive memory antibody responses following reinfection with serovar F

CT-specific antibody responses elicited at 1 and 4 weeks after rechallenge with CT serovar F were measured by antibody ELISA that included antibody standards. The results showed that at week 1 after reinfection, rVCG-MECA-immunized mice elicited robust systemic IgG2c antibody responses that were comparable to those in the live CT-immunized mice but significantly (*p* < 0.0001) higher compared to those in the PBS control mice irrespective of immunization route (**Figure 5A**). The systemic and mucosal IgA levels elicited following reinfection were comparable in all immunization and control groups (**Figure 5**). While both immunization routes induced IgG2c and secretory IgA antibody levels in vaginal secretions that were comparable with those in the live CT-immunized mice, the IM route induced significantly (*p* < 0.0001) higher IgG2c antibodies compared to those in the IN and PBS control mice (**Figure 5B**). These robust vaccine-induced antibody levels were sustained at week 4 following rechallenge (**Figures 5C, D**). The systemic IgG2c antibodies elicited in rVCG-MECA- and CT-immunized mice were comparable but significantly (*p* < 0.0001) higher than those elicited in PBS control immunized mice (**Figure 5C**). The IgG2c antibodies induced in the vaginal secretions of IN immunized mice 4 weeks after rechallenge were comparable to those of live CT-immunized mice, but significantly (*p* < 0.01) higher than those elicited in PBS control immunized mice (**Figure 5D**). Also, IM vaccine-immunized mice elicited significantly (*p* < 0.0001) higher IgG2c antibody levels than those elicited in IN vaccine- and PBS-immunized control mice after reinfection (**Figure 5D**).

**Figure 5:**
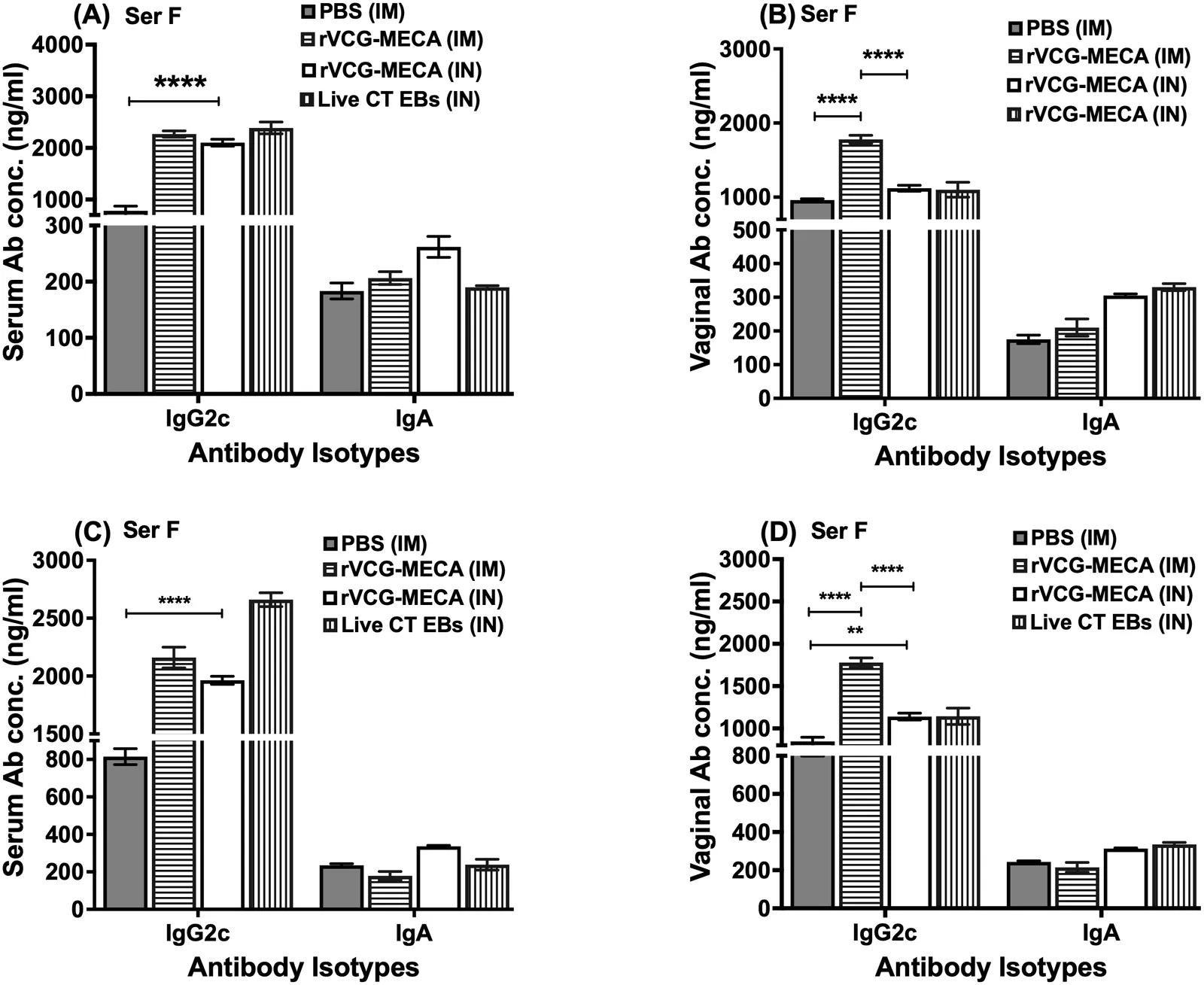
Induction of cross-reactive systemic and mucosal memory antibody responses after TC heterologous reinfection with CT Ser F. Groups of mice were immunized and boosted IM or IN as described in the Materials and Methods section. Four weeks after immunization, mice were challenged TC with 10^6^ IFU of live CT Ser F. Ten weeks after the primary challenge, the mice were rechallenged TC with the same dose. Serum and vaginal lavage samples were collected at weeks 1 **(A, B)** and 4 **(C, D)** following rechallenge. The amount of systemic and mucosal IgG2c and IgA memory antibodies elicited in serum (systemic) **(A, C)** and vaginal lavage (mucosal) **(B, D)** samples was assessed by standard antibody ELISA that included antibody standards. Results were generated simultaneously with a standard curve and display data sets corresponding to absorbance values as mean concentrations (ng/mL) ± SD for each experiment. The data are from one of three independent ELISAs with similar results. Differences between vaccine groups were compared by two-way ANOVA with Tukey’s post-multiple comparison test at **p < 0.01 and ****p < 0.0001.

### Induction of cross-reactive memory antibody responses following reinfection with serovar J

CT-specific antibody responses elicited after rechallenge with CT serovar J were also titrated by an antibody ELISA that included antibody standards. The cross-reactive systemic and mucosal IgG2c antibody responses elicited in rVCG-MECA-immunized mice were as robust as those in the live CT-immunized mice at weeks 1 and 4 after rechallenge (**Figure 6**). The systemic IgG2c antibody levels elicited at both time points following reinfection were significantly (*p* < 0.0001) higher than those in PBS controls, irrespective of immunization route (**Figures 6A, C**). Mice that were previously immunized IM elicited IgG2c antibody levels that were significantly (*p* < 0.05) higher in serum (**Figures 6A, C**) and vaginal secretions (**Figures 6B, D**) at both time points than those immunized IN. IgA antibodies elicited in serum at week 1 (**Figure 6A**) and in vaginal secretions at both time points (**Figures 6B, D**) were comparable in all immunization and control groups. However, at week 4 following reinfection, IgA antibody levels were significantly higher (*p* < 0.05) in serum of mice immunized IM compared to IN immunized mice (**Figure 6C**).

**Figure 6:**
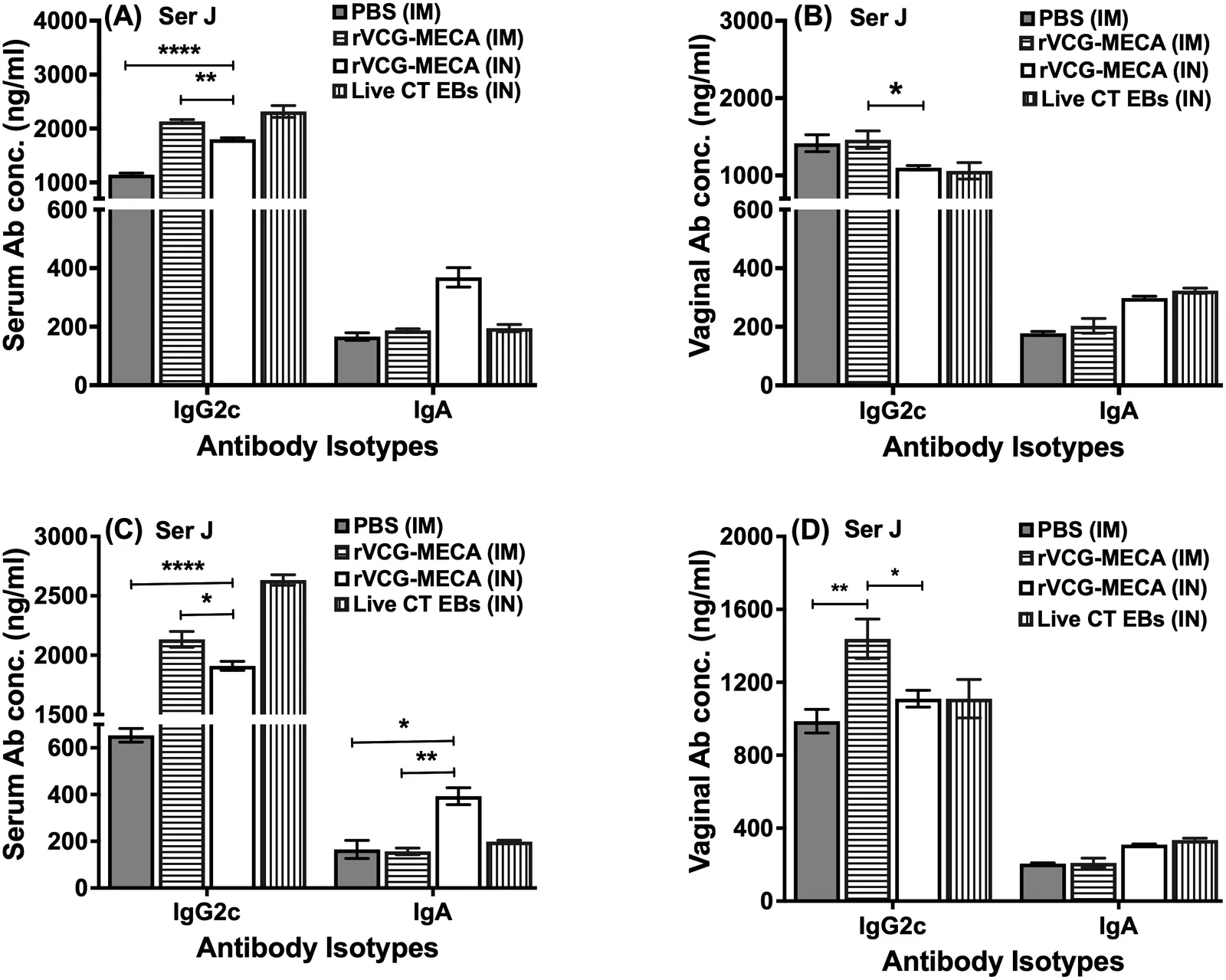
Cross-reactive systemic and mucosal memory antibody responses after TC heterologous reinfection with CT Ser J. Groups of mice were immunized and boosted IM or IN as described above. Four weeks after immunization, mice were challenged TC with 10^6^ IFU of live CT Ser J. Ten weeks after the primary challenge, the mice were rechallenged TC with the same dose. Serum and vaginal lavage samples were obtained at weeks 1 **(A, B)** and 4 **(C, D)** following rechallenge. The amount of systemic and mucosal IgG2c and IgA memory antibodies elicited in serum (systemic) **(A, C)** and vaginal lavage (mucosal) **(B, D)** samples was assessed by standard antibody ELISA that included antibody standards. Results were generated simultaneously with a standard curve and display data sets corresponding to absorbance values as mean concentrations (ng/mL) ± SD of three independent ELISAs. The data are from two independent ELISAs with similar results. Significant differences between groups were compared by two-way ANOVA with Tukey’s post-multiple comparison test at *p < 0.05, *p < 0.01, and ****p < 0.0001.

### Magnitude of antigen-specific Th1/Th2 cytokine responses induced following rechallenge of immunized mice with CT serovars F and J

To examine antigen-specific Th1/Th2 cell responses produced by immune T cells following rechallenge of immunized mice, pooled CD4^+^ T cells purified from SPL and genital tract DLN were restimulated with chlamydial antigen, and the magnitude of Th1/Th2 cytokines secreted in culture supernatants was analyzed. While vaccine-induced CD4^+^ T cells secreted high levels of the Th1 cytokines, IFN-γ and IL-12, in response to *in vitro* restimulation, levels of the Th2 cytokine, IL-4, and the anti-inflammatory cytokine, IL-10, were comparatively lower (**Figure 7**). Significantly higher (*p* < 0.0001) concentrations of IFN-γ and IL-12 were secreted by vaccine-induced splenic and genital DLN immune CD4^+^ T cells of mice reinfected with CT serovars F (**Figures 7A, B**) and J (**Figures 7C, D**) compared to those of PBS controls, irrespective of immunization route. In contrast, there was no difference in the levels of IL-4 and IL-10 produced by CD4 T cells from rVCG-MECA vaccine- and PBS control immunized mice, irrespective of challenge serovar (**Figures 7A–D**). The ratio between vaccine-induced IFN-γ and IL-4 concentrations was >20, irrespective of reinfecting CT Ser or immunization route. Collectively, these results demonstrate that immunization with rVCG-MECA induces cross-reactive, antigen-specific Th1-type cellular immune response following rechallenge with heterologous CT serovars.

**Figure 7:**
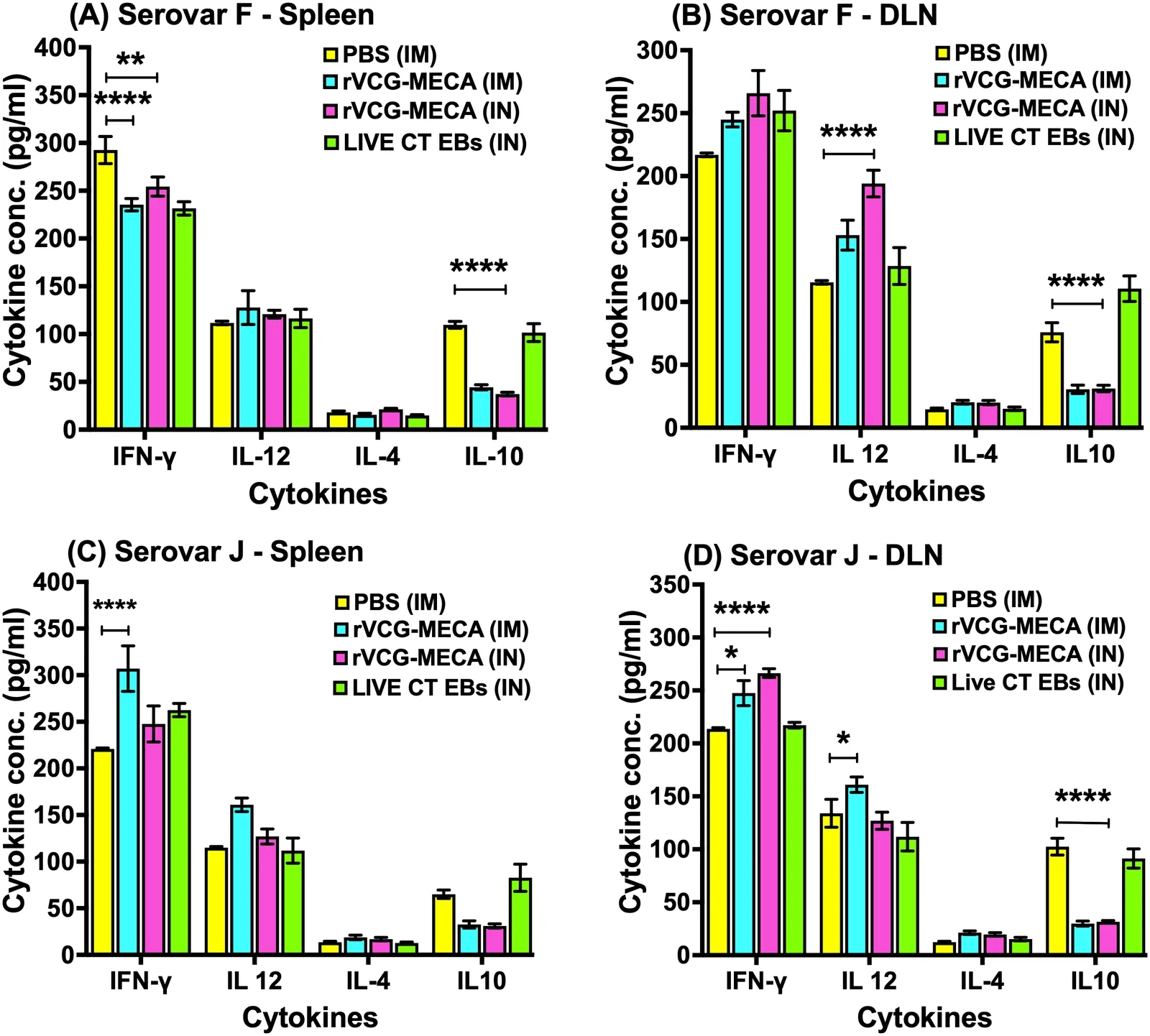
Induction of antigen-specific Th1/Th2 cytokine responses after reinfection with CT Ser F and J. Pooled CD4^+^ T cells were purified from the spleens **(A, C)** and genital tract DLN **(B, D)** of immunized and reinfected mice and were restimulated *in vitro* with chlamydial antigen. The amount of cross-reactive *Chlamydia*-specific Th1/Th2 cytokines contained in culture supernatants of stimulated CD4^+^ T cells was measured using the Bio-Plex cytokine assay kit. The concentration of the cytokines in each sample was obtained by extrapolation from a standard calibration curve generated simultaneously. Data were calculated as the mean values (± SD) for triplicate cultures for each experiment. The controls (cultures without antigen) did not contain detectable levels of cytokine and so the data were excluded from the results. The results are from two independent experiments and are shown as mean cytokine concentrations (pg/mL) ± SD. Significant differences between vaccine groups and between IFN-γ and IL-4 (Th1 and Th2 cytokines) were compared by two-way ANOVA with Tukey’s post-multiple comparison test at *p < 0.05, **p < 0.01, ****p < 0.0001.

### Induction of long-term cross-protection against rechallenge with live CT serovars F and J

Ten weeks after the primary challenge, the mice were rechallenged TC with 10^6^ IFUs of live CT serovar F or J and chlamydial burden was monitored by enumeration of chlamydial inclusions from cervicovaginal swabs. The result showed that immunized mice exhibited accelerated bacterial clearance, indicated by significant (*p* < 0.0001) reduction in the number of mice shedding chlamydiae and the number of chlamydial inclusions shed compared to PBS-treated controls following rechallenge (**Figure 8**). By day 9 after rechallenge, while almost all the rVCG-MECA- and live CT-vaccinated mice had cleared the infection, PBS-immunized mice were still shedding high numbers of chlamydiae, and by day 15 post rechallenge, all the rVCG-MECA-vaccinated mice had completely resolved the CT infection. These results reveal that immunization with the rVCG-MECA vaccine induces durable immunological memory resulting in accelerated clearance of infection following rechallenge, irrespective of route of immunization or challenge serovar.

**Figure 8:**
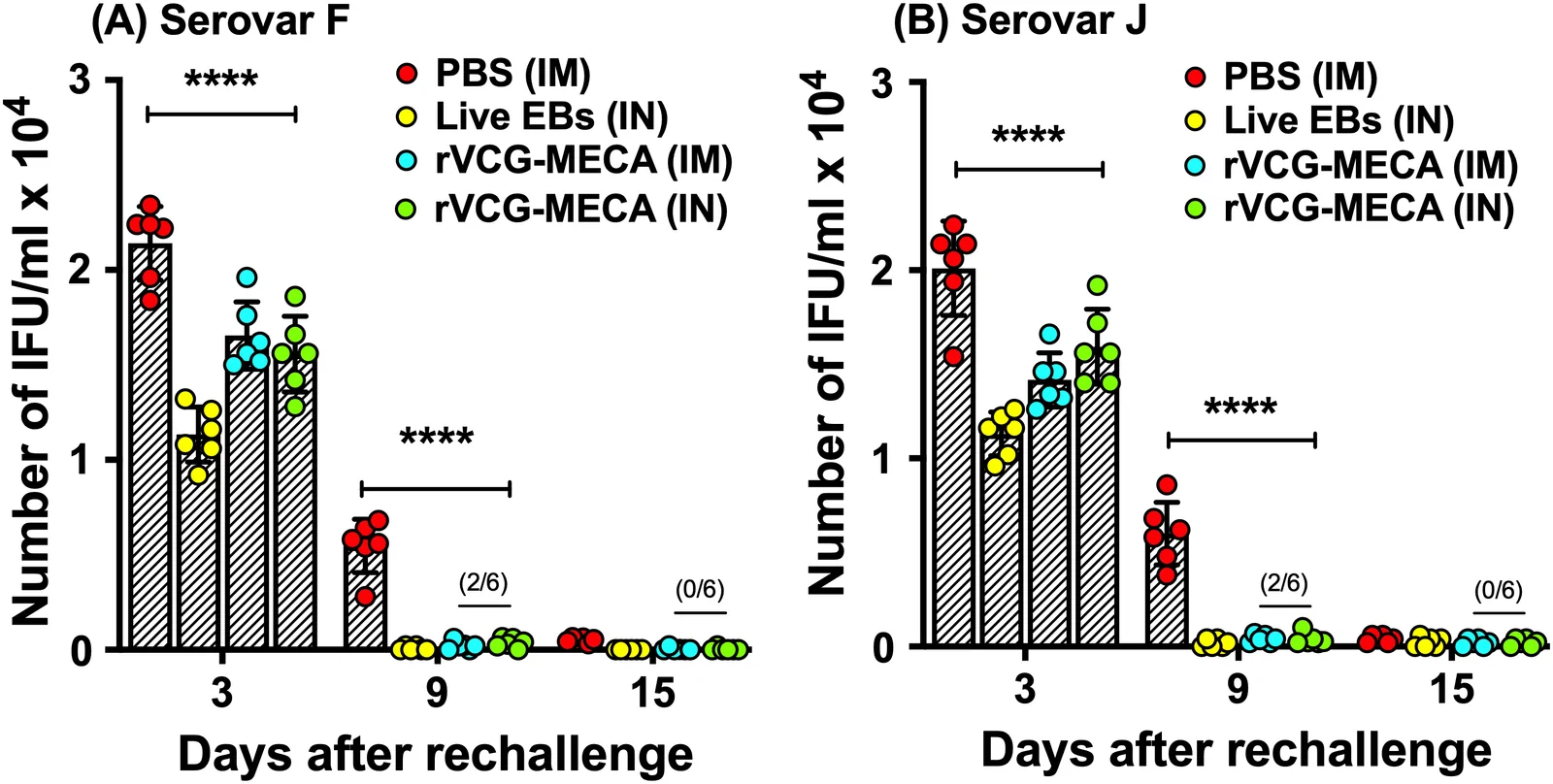
Long-term cross-protection of rVCG-MECA vaccine-immunized mice against heterologous reinfection with the CT serovars F and J. Groups of mice were immunized and boosted twice IM or IN as described. Cross-protection against heterologous reinfection with 10^6^ IFU of live CT Ser F **(A)** or J **(B)** was evaluated 10 weeks after the primary challenge as described above. CT burden was monitored by enumeration of chlamydial IFU from cervicovaginal swabs of mice every 3 days and the number of IFU/mL recovered was calculated. Each data point represents the individual number of recoverable IFU/mL from each mouse (distinct colored circle) and the mean IFU for each group (represented by each column). Numbers in parentheses represent the number of mice with positive cultures at each time point. The differences between vaccine groups were compared by two-way ANOVA with Tukey’s post-multiple comparison test at ****p < 0.0001.

## Discussion

We previously reported that systemic (IM) or mucosal (IN) delivery of rVCG-MECA elicited robust cellular and long-lasting humoral immune effectors that protected mice against TC challenge infection with the homologous CT Ser D (cluster 1) (Medhavi et al., 2025). In the present study, we evaluated if immunization with the rVCG-MECA vaccine will induce the production of serum antibodies with high binding capacity to the heterologous Ser F and J as an indicator of the ability of the vaccine to induce cross-reactive immunological memory. Memory responses are characterized by the production of high-avidity antibodies, and generation of antibody avidity maturation is indicative of the induction of immunological memory and a T-cell-dependent immune response (Longworth et al., 2002; Vermont et al., 2002). Evaluation of antibody avidity showed that while the avidity of serum IgG and IgG2c antibodies elicited following IM immunization with rVCG-MECA was moderate, the avidity of these antibodies was comparatively high following IN immunization as we previously reported (Richardson et al., 2025). These findings also confirm the results of a previous study indicating that the Th1-associated IgG2c antibody isotype elicited following IN immunization with rVCG-MECA has higher binding capacity compared to those induced by IM immunization (Medhavi et al., 2025). High-avidity antibodies have been demonstrated to have better functional capacities in bacterial killing and inducing protection against experimental infection than antibodies of low avidity (Usinger and Lucas, 1999). Taken together, these results indicate the ability of rVCG-MECA to induce durable immunological memory upon challenge.

We also sought to evaluate the ability of rVCG-MECA to confer cross-protection against primary and secondary infection with the heterologous CT Ser F and J. These serovars were selected for this study because they are the senior members of clusters 2 (Ser F) and 3 (Ser J) of the 11 oculogenital CT Ser and have distinct antigenic profiles in clinical oculogenital infections compared to the cluster 1 members (Ser B, D, and E) (Barnes and Cathcart, 1998; Lanfermann et al., 2021). This provides a stringent test for evaluating the ability of the rVCG-MECA vaccine to induce cross-protective immunity against heterologous chlamydial serovars. Based on the demonstrated within-cluster cross-Ser protection (Tuffrey et al., 1992; Ramsey et al., 1999), if immunization with rVCG-MECA protects against serovar F, it will protect against Ser G, the other member of cluster 2. Similarly, if the vaccine protects against serovar J, it will protect against all members of cluster 3 (Ser A, C, H, I, and K), indicating its ability to protect against all 11 oculogenital CT Ser. Vaccine efficacy evaluation revealed that mice immunized with rVCG-MECA had considerably reduced chlamydial burden as early as day 3 through day 15, culminating in successful resolution by day 21 after a high primary challenge dose (10^6^ IFU). The level of protection was comparable to that conferred by immunization with live CT EBs (positive control).

The rapid reduction in genital chlamydial burden and the shortened duration of CT shedding upon rechallenge with a high dose of live CT suggests that immunization with rVCG-MECA generates durable immunological memory capable of mediating effective recall responses upon reinfection. Together, these findings are consistent with the core requirement for a translational CT vaccine, which is the ability to confer protection that is not restricted to a single serovar and that persists beyond the immediate postimmunization window. The ability of rVCG-MECA to protect against all eight urogenital and three trachoma Ser (A, B, and C) will be confirmed in future studies. Although immunization via parenteral routes have been shown to be effective against genital challenge with CT (Eko F. et al., 2011; Bøje et al., 2016; Medhavi et al., 2025), a previous report indicated that vaccination regimens involving mucosal priming confer superior protection following TC challenge due to the induction of tissue-resident memory (TRM) T cells in the genital tract that respond rapidly to infection (Stary et al., 2015). However, recent reports have demonstrated that parenteral immunization is equally effective at inducing effective protection following TC challenge (Nguyen et al., 2020; Medhavi et al., 2025). Our findings demonstrating the ability of rVCG-MECA to confer significant protection against TC infection following systemic and mucosal immunization confirms these recent reports. It is likely that rVCG-MECA vaccine-induced protection is mediated by the induction of TRM T cells in addition to other immune effectors in the genital mucosa. This is part of the subject of ongoing studies.

The ability of rVCG-MECA to induce cross-reactive memory antibody responses following reinfection of IM or IN immunized mice with the heterologous CT Ser F and J was also evaluated. The rapid induction of high levels of mucosal and systemic IgG2c and IgA antibodies as early as week 1 that remained high at week 4 after rechallenge indicates that immunization with rVCG-MECA leads to the development of B-cell memory that can be recalled even after rechallenge. Our results showing higher levels of the Th1-associated IgG2c compared to IgA antibodies in vaginal secretions confirm the results of our previous studies (Pais et al., 2017; Pais et al., 2019; Medhavi et al., 2025) and parallel findings from human studies showing that IgG but not secretory IgA is the major immunoglobulin present in female genital tract secretions (Mestecky et al., 2005; Agrawal et al., 2009). These findings indicate that both IM and IN immunization with rVCG-MECA induces robust cross-reactive memory antibody responses that can be recalled in both systemic and mucosal immune compartments following reinfection. The route-dependent skewing of the Th1-associated IgG2c and mucosal IgA antibodies elicited in vaginal secretions of reinfected mice following IM and IN immunization confirms the results of our previous studies showing the induction of higher levels of vaginal IgG2c and IgA antibodies following IM and IN rVCG-MECA immunization, respectively (Medhavi et al., 2025). While the precise role of antibodies in protection against primary CT infection is still unraveling, we had previously reported the association of the Th1-associated IgG2c antibody isotype with chlamydial immunity (Moore et al., 2003). Also, a recent report indicates that genital mucosal secretory IgA elicited following peroral immunization with inactivated or live *C. muridarum* protects against primary genital chlamydial infection (Shillova et al., 2020). It is therefore plausible that the major role of antibodies in protection may be to foster a rapid memory Th1 cell response during reinfection, which is essential for bacterial containment in the genital mucosa (Igietseme et al., 2004; Morrison and Morrison, 2005; Gann et al., 2022).

CT infection-induced immunity in mice is mediated primarily by IFN-γ-secreting CD4^+^ Th1 cells and accessory antibodies (Loomis and Starnbach, 2002; Morrison and Caldwell, 2002; Igietseme et al., 2004; Bøje et al., 2016; Johnson and Brunham, 2016; Gann et al., 2022). Following mouse genital tract infection, CD4 T cells and B cells tend to cluster together in the infected tissue, forming immune cell aggregates where they can effectively coordinate an immune response against the pathogen, with CD4 T cells playing a key role in activating B cells to produce antibodies specific to the infection (Murray and McKay, 2021). These findings have furnished important immunologic correlates for vaccine testing and evaluation, suggesting that vaccines capable of boosting IFN-γ-producing CD4 Th1 and B cells in the mouse genital tract are likely to lead to induction of optimal protective immunity. However, recent reports indicate that classical T-bet-expressing Th1 cells are not essential for clearance of primary genital *C. muridarum* infection in mice (Rixon et al., 2022; Rixon et al., 2024). Whether this is applicable to clearance of CT genital infection following vaccine-induced immune responses is yet to be determined. Moreover, a previous report showed that mature follicular T helper (Tfh) cells produce IFN-γ without expressing the Th1 transcription factor T-bet and demonstrated that almost all the IFN-γ-producing Tfh cells have previously expressed T-bet (Fang et al., 2018). Following rechallenge, antigen restimulation of CD4^+^ T cells from spleen and genital tract DLN produced significantly higher IFN-γ and IL-12 levels compared to IL-4, indicating a Th1-type cellular recall response similar to that reported following rVCG-MECA immunization (Medhavi et al., 2025). This immunological profile is aligned with established mechanisms of resistance to *Chlamydia* infection, where IFN-γ mediates key antimicrobial effects and IL-12 supports Th1 polarization and memory formation. Experimental evidence across murine models has repeatedly shown that impairments in the IL-12/IFN-γ axis delay bacterial clearance and worsen disease outcomes, underscoring the central role of this pathway in protection (Del Rio et al., 2001; Brunham et al., 2005; Gondek et al., 2009; Jupelli et al., 2010; Rixon et al., 2022). The comparatively limited IL-4 and IL-10 responses relative to IFN-γ/IL-12 are consistent with a response profile that favors effective control of an intracellular pathogen rather than a Th2-skewed program associated with reduced clearance in multiple models (Brunham et al., 2005; Gondek et al., 2009). Although rVCG-MECA vaccine-induced secretion of IL-17 was not assessed in this study, we had previously reported the induction of IFN-γ and IL-17 by immune CD4 T cells purified from the spleens of rVCG-PmpD/PorB-immunized mice (Pais et al., 2017). Together, the combined humoral and cellular immune signatures induced by rVCG-MECA, involving systemic and mucosal IgG2c and IgA antibody and IFN-γ/IL-12-dominated cytokine recall responses, support a mechanism of protection consistent with our current understanding of the immunobiology of *Chlamydia*.

A significant feature of the rVCG-MECA vaccine is its multiepitope design, which targets conserved antigenic regions of multiple antigens (Tanner et al., 2024; Medhavi et al., 2025) rather than relying on a single dominant antigen. Historically, serovar variability has been a central challenge for CT vaccine development, particularly for strategies heavily dependent on a single whole antigen, such as MOMP (Kari et al., 2011; de la Maza et al., 2017). By incorporating multiple immunogenic components, rVCG-MECA is capable of mitigating serovar-specific escape and broadening immune recognition, as indicated by its ability to confer cross-serovar protection against challenge with serovars F and J. In this context, our findings parallel emerging clinical and preclinical evidence supporting multiantigen vaccine approaches. For example, CTH522/CAF^®^01, a multisubunit vaccine comprising MOMP variable domains from multiple CT serovars has demonstrated safety and induction of immune signatures consistent with cross-serovar protection in early-stage studies, underscoring the feasibility of multiantigen strategies for human translation (Olsen et al., 2024). While direct comparisons should be made cautiously due to differences in antigen composition, adjuvants, and immunization routes, the convergence of immune patterns supports the broader premise that multiantigen constructs can overcome serovar-driven limitations (Eko F. O. et al., 2011; Pais et al., 2017).

In conclusion, immunization with rVCG-MECA induced significant cross-serovar protection against CT serovars F and J relative to PBS controls and established durable immunological memory, as indicated by the production of high-avidity antibodies and accelerated bacterial clearance upon secondary challenge. Both IM systemic and IN mucosal immunization routes conferred significant protection against primary and secondary challenge, while promoting distinct yet complementary immune profiles. It should be noted that the protection observed following reinfection is associated with the humoral and cellular immune effectors induced by both vaccination and prior primary and secondary infections. The mucosal IgA and systemic IgG2c responses coupled with the Th1-dominant (IFN-γ versus IL-4) recall responses support a protective mechanism consistent with established requirements for anti-chlamydial immunity. The multiepitope design of rVCG-MECA, targeting conserved immunogenic regions of multiple antigens, underlines its ability to overcome serovar-specific limitations that have challenged prior vaccine efforts. Together, these findings support rVCG-MECA as a promising multiantigen vaccine capable of inducing broad and durable protective immunity against genital CT infection.

## Data Availability

The original contributions presented in the study are included in the article/Supplementary Material. Further inquiries can be directed to the corresponding author.
